# The effect of solvent on reactivity of the Li_2_S–P_2_S_5_ system in liquid-phase synthesis of Li_7_P_3_S_11_ solid electrolyte

**DOI:** 10.1038/s41598-021-00662-3

**Published:** 2021-10-26

**Authors:** Hirotada Gamo, Atsushi Nagai, Atsunori Matsuda

**Affiliations:** Department of Electrical and Electronic Information Engineering, Toyohashi University of Technology, 1-1 Hibarigaoka, Tempaku-cho, Toyohashi, Aichi 441-8580 Japan

**Keywords:** Energy storage, Energy science and technology, Materials science

## Abstract

Synthesis technology for sulfide-based solid electrolytes based on liquid-phase processing has attracted significant interest in relation to achieving the optimal design for all-solid-state batteries. Herein, guidelines to solvent selection for the liquid-phase synthesis of superionic conductor Li_7_P_3_S_11_ are described through systematic examination. 70Li_2_S–30P_2_S_5_ system, a source of Li_7_P_3_S_11_, is treated via a wet chemical reaction using eight organic solvents with different physical and chemical properties (i.e., dielectric constant, molecule structure, and boiling point). We reveal that the solvent’s polarity, characterized by the dielectric constant, plays an important role in the formation of crystalline Li_7_P_3_S_11_ via wet chemical reaction. In addition, acetonitrile (ACN) solvent with a high dielectric constant was found to lead to high-purity crystalline Li_7_P_3_S_11_ and intrinsically high ionic conductivity. Further, solvents with a high boiling point and ring structures that cause steric hindrance were found to be unfavorable for the wet chemical synthesis of Li_7_P_3_S_11_ solid electrolyte. Overall, we demonstrate that ACN solvent is the most suitable for the liquid-phase synthesis of a crystalline Li_7_P_3_S_11_ solid electrolyte with high purity based on its dielectric constant, molecular structure, and boiling point.

## Introduction

Herein, we investigate solvent selection focusing on three solvent physical properties, namely, dielectric constant, molecule structure, and boiling point. We focus on the effects different solvents have on increasing the ionic conductivity of the inorganic Li_7_P_3_S_11_ electrolyte to contribute to the reactivity of 70Li_2_S–30P_2_S_5_ during liquid-phase synthesis. Even now, the rapid growth of lithium-ion batteries has raised safety concerns regarding commercial lithium-ion batteries with liquid electrolytes. In particular, the use of flammable organic liquid electrolytes can cause leakage from and ignition of the electrolytes^1,2^. Replacing the organic liquid electrolyte with a solid electrolyte will ensure excellent battery safety. All-solid-state lithium batteries with inorganic solid electrolytes that contain crystalline Li_7_P_3_S_11_ are one of the most promising energy storage technologies due to their high safety and high energy density, especially for electric vehicles^3^. Among solid electrolytes, sulfide-based lithium-ion solid electrolytes exhibit high ionic conductivity compared with liquid electrolytes. In contrast to oxide solid electrolytes, they also have a mechanically soft nature, which benefits their physical interaction with active materials^4^. These materials generally contain Li_10_GeP_2_S_12_ (σ_25_ = 12 mS cm^–1^)^5^, Li_7_P_3_S_11_ (σ_25_ = 17 mS cm^–1^)^6^, and Li_6_PS_5_Cl argyrodites^7,8^.

Sulfide materials are prepared under an inert atmosphere because of their moisture-sensitive. Manufacturing of sulfide-based all-solid-state batteries is subjected to the severe limitation that poses major technical challenges^9^. Commercialization of all-solid-state batteries required a promising process technology toward scalable manufacturing of solid electrolytes and the mixing of electrode composites containing the solid electrolytes, cathode active materials, and conductive additives^10,11^. For the most part, the synthesis methods for sulfide solid electrolytes can be divided into three categories: (i) solid reaction methods, (ii) mechanochemical reaction methods, and (iii) wet chemical methods. Wet chemical methods are the most effective for obtaining nanosized solid electrolytes, making them advantageous for homogeneous compounding with the cathodic electrode. In addition, wet chemical methods are suitable for large-scale synthesis at low reaction temperatures^12^. Liu et al. have reported that a wet chemical method based on tetrahydrofuran (THF) solvent is effective for the preparation of *β*-Li_3_PS_4_, a typical sulfide electrolyte^13^. In addition, Li_2_S–P_2_S_5_ solid electrolytes have been synthesized in organic solvents, such as propionate (EP), butyl acetate (BA)^14^, and acetonitrile (ACN). Recently, Yamamoto et al. demonstrated the effect of solvent on the ionic conductivity of Li_3_PS_4_ synthesized using a liquid-phase process^14^. The ionic conductivity of Li_3_PS_4_ prepared from a liquid phase was found to have a strong correlation with the polarity, ${\delta }_{p}$, of the solvent, reaching 5.09 × 10^–4^ S cm^–1^ at room temperature when using BA with a low polarity. This value is consistent with the conductivity of a sample obtained via ball milling. In 2012, Li_7_P_3_S_11_ was synthesized as a high lithium-ion conductor in dimethyl ether (DME) solvent with a maximum ionic conductivity of 2.7 × 10^–4^ S cm^–1^ at room temperature^15^. Following this, Li_7_P_3_S_11_ was prepared using ACN, THF, and anisole solvents; the highest ionic conductivity was achieved using ACN solvent^16,17^. A wet chemical reaction mechanism for the formation of Li_7_P_3_S_11_ phase in ACN has also been proposed. It was found that two phases, soluble Li_2_S–P_2_S_5_ and solvated Li_3_PS_4_, could be converted to Li_7_P_3_S_11_ in the desolvation process during heat treatment^18,19^. Several research groups have also focused their efforts on the synthesis of Li_7_P_3_S_11_ based on ACN solvent^20–24^. Xu et al. observed differences between Li_7_P_3_S_11_ synthesized from THF and ACN solvents and suggested that the steric hindrance (ring or short-chain structure) of these solvent molecules results in the presence of residue molecules in the solid electrolyte^16^. However, due to the absence of systematic studies, no leading theory on the effects of solvent on the synthesis of highly conductive crystalline Li_7_P_3_S_11_ phase has been developed.

In this work, we investigated the effect of solvent on reactivity in the formation for Li_7_P_3_S_11_ solid electrolytes. In particular, we employed 1,4-dioxane (Dox), carbon disulfide (CS), tetrahydropyran (THP), nitromethane (NM), ACN, furfural (FF), succinonitrile (SN), and ethylene carbonate (EC) reaction medium. Using organic solvents with a high dielectric constant led to high reactivity in the Li_2_S–P_2_S_5_ system, forming high-purity crystalline Li_7_P_3_S_11_ from liquid-phase synthesis. However, all solvents with a high dielectric constant except for ACN underwent a side reaction with the Li_2_S–P_2_S_5_ system. Furthermore, they are not suitable for solvent removal via a drying process due to their high boiling point. Here, we demonstrate that ACN solvent is the most suitable solvent for the formation of Li_7_P_3_S_11_ solid electrolyte due to its high dielectric constant, linear structure, and low boiling point.

## Results and discussion

### Chemical, structural, and microstructural properties

**Figure 1:**
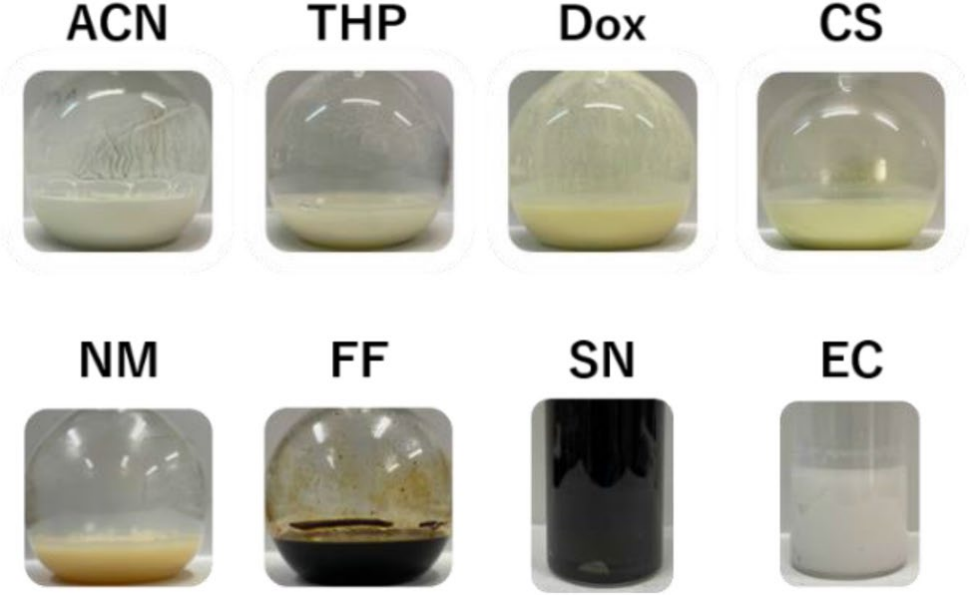
Photographs of 70Li_2_S–30P_2_S_5_ mixtures after stirring for 3 days in ACN, THP, Dox, CS, NM, FF, AN, and EC solvents.

**Figure 2:**
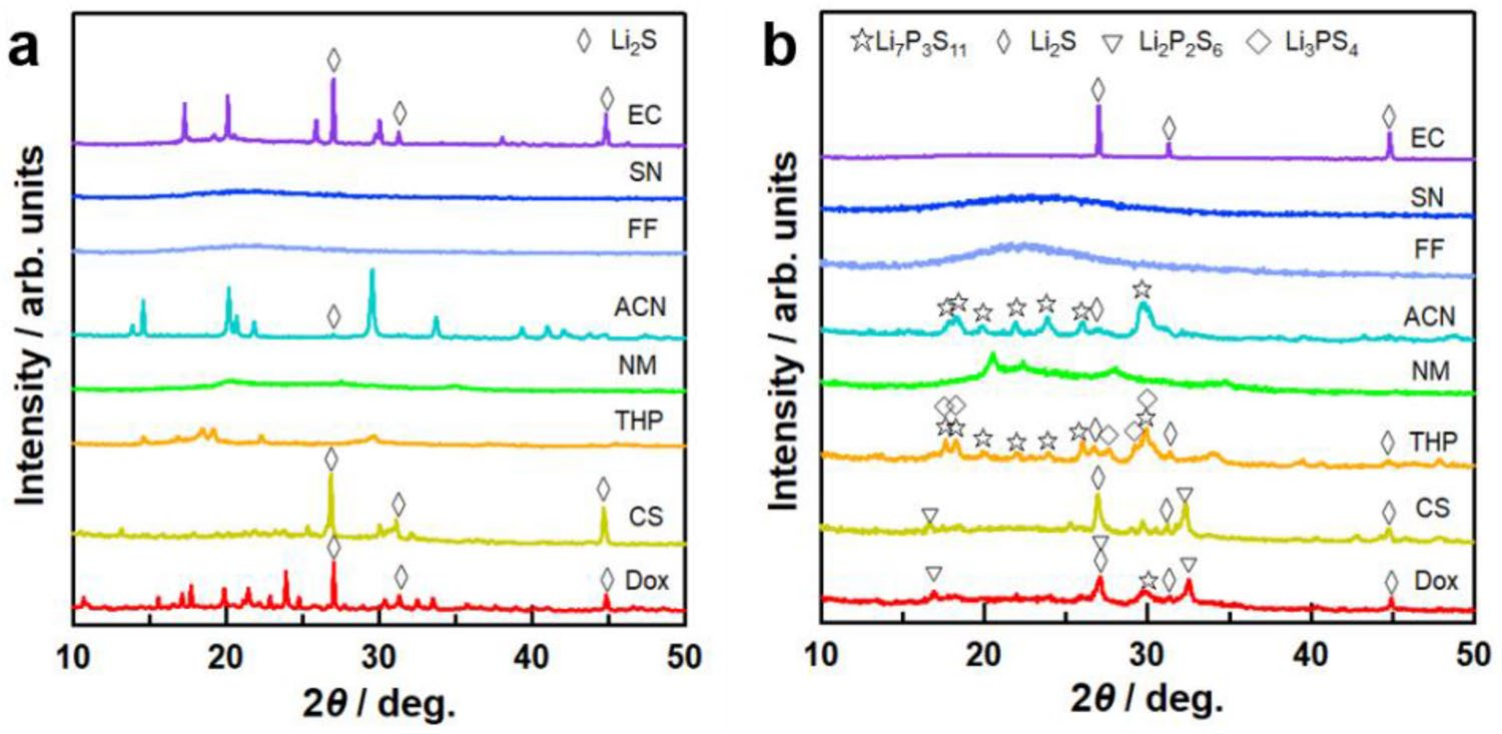
XRD patterns of (**a**) precursors after drying under vacuum and (**b**) 70Li_2_S–30P_2_S_5_ solid electrolytes synthesized using each solvent.

Figure 1 shows the suspensions for Li_7_P_3_S_11_ with each organic solvent after stirring at 50 °C for 3 days. The color of the suspension varied depending on the solvent. The suspensions with ACN and THP were milky white and off-white, respectively. Chemical reactivity in organic solvents generally depends on the bond polarity between solvent molecular and ionic species^25^. In particular, the dielectric constant and donor number (DN) of the organic solvent play a crucial role in the formation of a solvating complex. For instance, ACN molecules have stronger interactions with Li ions due to their high dielectric constant, as outlined in Table 1. In addition, the coordination of ACN with cations (in this case Li ions) is less affected by steric hindrance because of the linear structure^26^. The strong interaction between Li ions and ACN indicates their shorter bond length relative to those between Li ions and other solvents, resulting in the white color of the suspension, which is the typical color of the Li_3_PS_4_ complex. THP solvent also demonstrated a lower dielectric constant and steric hindrance induced by its cyclic structure, leading to a weaker interaction with Li ions. Using Dox, CS_2_, and NM as the reaction media resulted in a yellowish-white suspension, indicating poor reactivity to Li_3_PS_4_. The mixture of starting materials including FF solvent immediately became discolored (black) after the addition of the solvent, indicating the occurrence of a side reaction between the FF solvent molecules and the the Li_2_S–P_2_S_5_ system. In addition, the suspension with SN solvent gradually became black during stirring, probably resulting in the relatively strong cross-interaction between Li ions and the two CN groups in SN. Figure 2a,b show the X-ray diffraction (XRD) patterns of the precursors and samples, respectively, following heat treatment. The crystal structures of the precursors varied depending on the solvent due to the differences in polarity and structure of the solvent molecules. The XRD pattern of the precursor containing ACN solvent is consistent with that of Li_3_PS_4_$\cdot$ACN, as reported by Calpa et al^27^. At the same time, the Li_7_P_3_S_11_ precursor involves two phases of the Li_2_S$\cdot$P_2_S_5_ and Li_3_PS_4_ complexes^18^. According to a recent study, following a drying process at 100 °C, Li_2_S$\cdot$P_2_S_5_ with ACN exhibits a halo-shaped XRD pattern originating from its amorphous structure^28^. Therefore, the Li_7_P_3_S_11_ precursor with ACN solvent contains crystalline Li_3_PS_4_$\cdot$ACN and amorphous Li_2_S$\cdot$P_2_S_5_. Residue Li_2_S was observed in the precursors with ACN, THP, CS, and Dox solvents. The intensity of the peak corresponding to residual Li_2_S in the 70Li_2_S–30P_2_S_5_ solid electrolyte decreased in relation to increases in the dielectric constant of the solvent. Interestingly, the precursor with EC solvent retained a large amount of unreacted Li_2_S without being affected by the high polarity of the EC solvent. It has also been reported that Li-sulfur batteries using EC-based liquid electrolytes suppress the dissolution of lithium polysulfides generated during the electrochemical cycling process^29^. This indicates that EC solvent has low solubility for ionic species containing sulfur, which is consistent with the low reactivity of the EC solvents with Li_2_S and P_2_S_5_ observed in our study.

**Table 1:** Physical and chemical properties of ACN, THP, Dox, CS, NM, FF, AN, and EC solvents.

Solvent	Structure	Boiling point ($^\circ$C)	Dielectric constant
Dox		101	2.2^22^
CS		46	2.63^24^
THP		88	5.5^25^
NM		101	35.9^22^
ACN		82	38^22^
FF		162	42^26^
SN		267	55^27^
EC		248	89.1^22^

Li_7_P_3_S_11_ crystalline phase was successfully synthesized in the samples with ACN, THP, and Dox solvent following heat treatment. The intensity of the first peak originated from Li_7_P_3_S_11_ crystal phase at 29.7 degrees increased in the order of Dox, THF, and then ACN solvents, which showed value of 232, 316, and 386, respectively. This observation suggests that higher dielectric constant of the solvent results in higher purity of crystalline Li_7_P_3_S_11_. In the case of the samples containing THP and Dox, Li_3_PS_4_ and Li_2_P_2_S_6_ crystalline phases remained following heat treatment. This is consistent with the trend observed in Li_7_P_3_S_11_ synthesized from the liquid phase in ACN and THF solvents demonstrated in a previous study^16^. The sample with NM solvent involved an unknown phase, while the samples containing FF and SN exhibited amorphous structure before and after heat treatment at 270 °C. Considering the color of the precursor suspension and the XRD results, it is likely that the FF and SN solvent molecules caused a side reaction with the initial materials. The aldehyde was also activated by the action of Lewis acid as a catalyst. FF solvent is an aromatic compound with an aldehyde group, which indicates that the side reaction was caused by the coordinate bond between Li ions and negatively charged oxygen molecules within the carboxyl group. In addition, Li ions attacked the oxygen molecules of the cyclic compound within the FF solvent molecules, which is associated with ring opening and a chain of side reactions.

**Figure 3:**
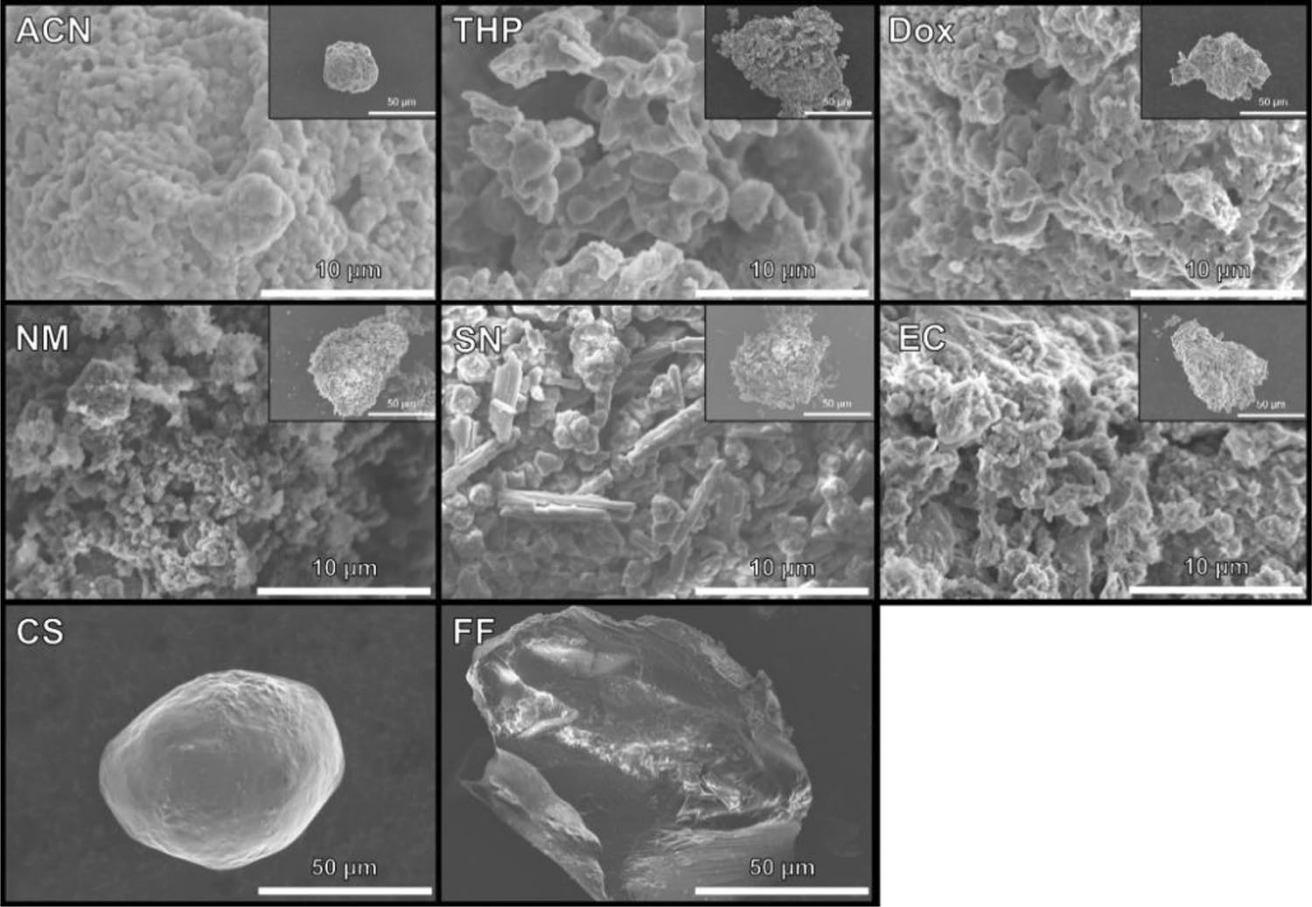
SEM images of 70Li_2_S–30P_2_S_5_ synthesized via ACN, THP, Dox, NM, SN, EC, CS, and FF solvents.

Figure 3 shows field emission scanning electron microscope (FE-SEM) images of 70Li_2_S–30P_2_S_5_ solid electrolytes prepared via each solvent. In all samples, the secondary particles ranged in diameter from 50 to 100 μm. The microstructures of the samples containing Li_7_P_3_S_11_ crystal phase created via ACN, THP, and Dox exhibited intriguing textures, including one resembling a broccoli crown. The microstructure of the sample prepared via ACN solvent involved uniform pseudo-spherical primary particles of less than 1 μm, and each particle was aggregated and connected with the others. It is clear that the homogeneous Li_7_P_3_S_11_ was produced through a solid reaction. In the case of THP and Dox, the samples formed primary particles with nonuniform shapes, exhibiting rougher particles than the sample created via ACN solvent. In the case of NM, SN, and EC, the microstructures exhibited a very rough particle surface. Monolithic particles of greater than 50 µm were observed in the samples synthesized via CS and FF, which means that the residue included coordinated complexes and compounds generated from a side reaction, as shown in the thermogravimetry/differential thermal analysis (TG–DTA) results.

### Desolvation process for Li_7_P_3_S_11_ solid electrolyte formation

**Figure 4:**
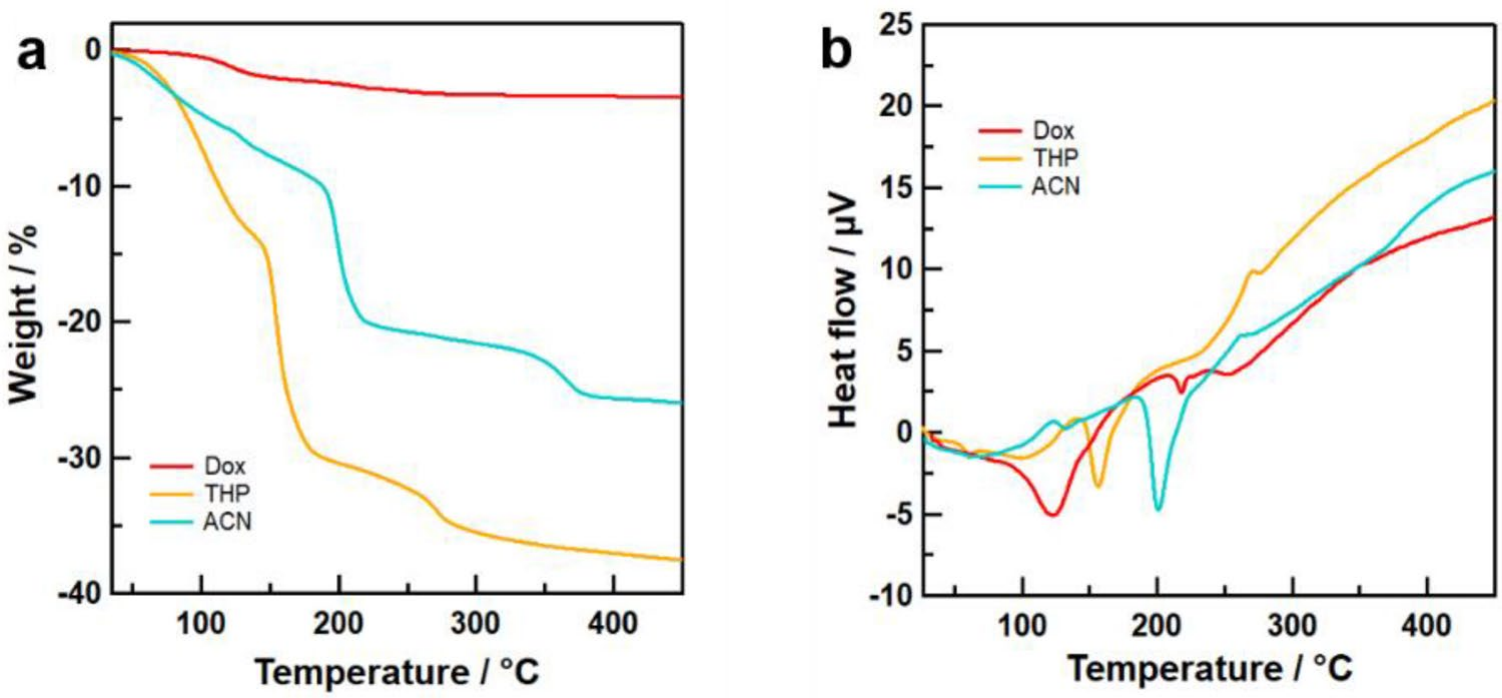
(**a**) TGA curves and (**b**) DTA curves of 70Li_2_S–30P_2_S_5_ precursors synthesized using Dox, THP, and ACN as solvents.

Figure 4a,b display the TGA and DTA curves, respectively, for the precursors in Dox, THP, and ACN solvents. The precursor powders in ACN and THP both exhibited three steps of weight loss. Several studies have demonstrated that forward DTA scans of Li_3_PS_4_ complexes containing organic solvent exhibit large endothermic peaks^13,14^. Further, the decomposition reaction of Li_7_P_3_S_11_ to Li_4_P_2_S_6_, Li_3_PS_4_, and sulfur occurs at a temperature higher than 280°C^30,31^. Thus, the first step corresponded to the evaporation of solvent molecules coordinated to Li_2_S–P_2_S_5_, the second step corresponded to the evaporation of solvent molecules coordinated to Li_3_PS_4_, and the third step corresponded to the evaporation of sulfur with the decomposition of Li_7_P_3_S_11_. The heat treatment temperature for the formation of Li_7_P_3_S_11_ solid electrolytes was determined from the TG–DTA results. The precursors with Dox, THP, and ACN involved the solvent molecule at 3.0 wt%, 32.3 wt%, and 20.7 wt%, respectively. The respective mole ratios of the coordinated solvent molecules of Dox, THP, and ACN in the precursor were 0.17, 2.73, and 3.14 against 70Li_2_S–30P_2_S_5_. The extent of the weight loss is not correlated with the boiling point of the solvent. The precursors with Dox, THP, and ACN solvents exhibited endothermic peaks at 120 °C, 150 °C, and 200 °C, respectively. These observations indicate that the strength of the chemical interaction between the Li_2_S–P_2_S_5_ system and solvent molecules within the precursor structure increases in the order of Dox, THP, and then ACN. This reflects solvent polarity, characterized by the dielectric constant. In the case of the precursors containing the NM, FF, and SN solvents, continuous weight loss during the annealing process was observed up to 450 °C (Figure S1). This experimental result indicates that these solvent molecules remain following heat treatment at 270 °C. It should be noted that these observations may be influenced by the evaporation of sulfur within 70Li_2_S–30P_2_S_5_ during heat treatment.

### Ionic conductivity of 70Li_2_S–30P_2_S_5_ system

**Figure 5:**
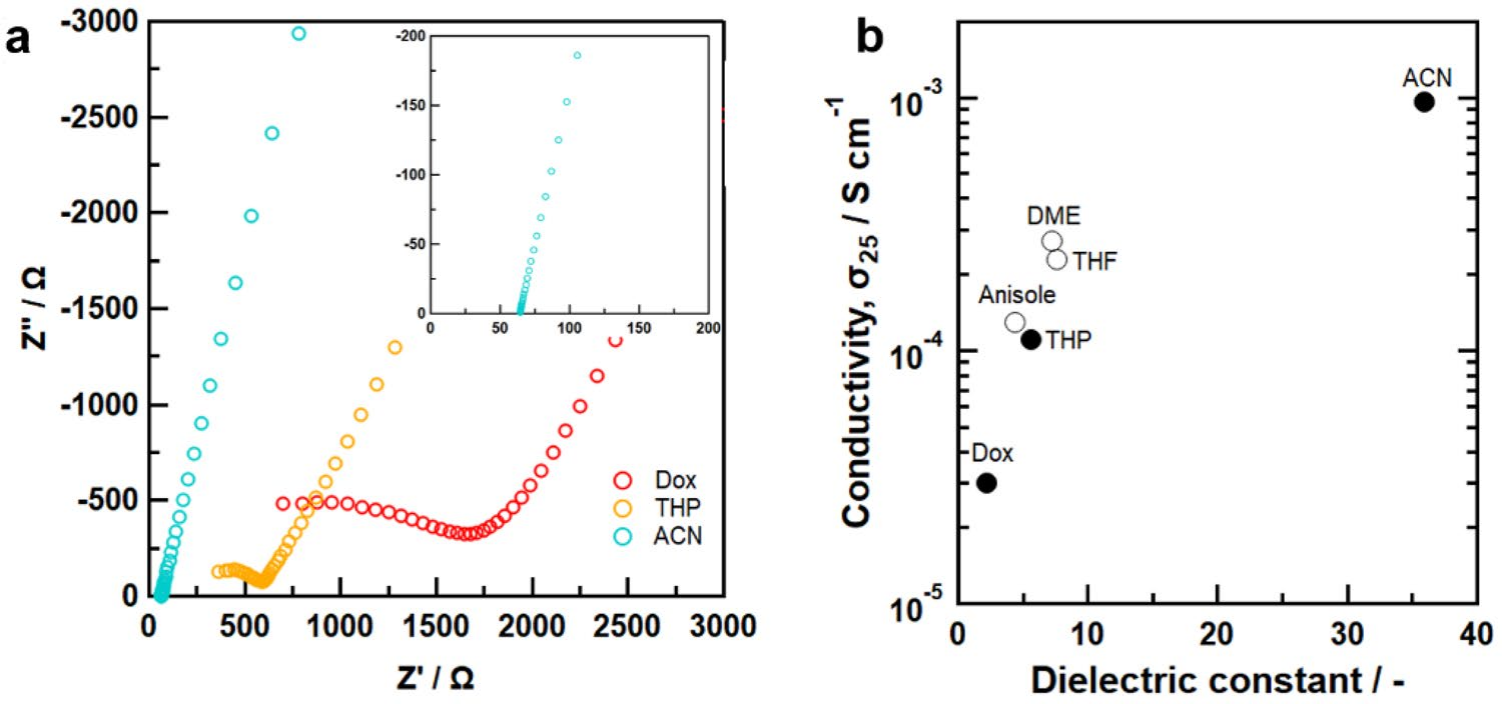
(**a**) Nyquist plot and (**b**) ionic conductivity at room temperature for 70Li_2_S–30P_2_S_5_ system formed via Dox, THP, and ACN solvents as a function of dielectric constant together with those of 70Li_2_S–30P_2_S_5_ system formed via other solvents^12–14^. The white circles indicate reference values.

Figure 5a shows Nyquist plots of the 70Li_2_S–30P_2_S_5_ solid electrolytes synthesized using Dox, THP, and ACN. The total resistance, including the bulk and grain boundary contributions, was determined by the real-axis intercept at high frequency. The resistance of solid electrolytes decreased in the order of Dox, THP, and then ACN. Figure 5b shows the calculated conductivities as a function of the solvent’s dielectric constant. These results revealed the correlation between the dielectric constant of the solvent and the ionic conductivity of the obtained solid electrolytes. The 70Li_2_S–30P_2_S_5_ solid electrolytes synthesized using ACN solvent exhibited the highest ionic conductivity among the prepared samples of 0.8 mS cm^–1^ at room temperature. The ionic conductivity for 70Li_2_S–30P_2_S_5_ system depends on the crystallinity, and highly crystalized 70Li_2_S–30P_2_S_5_ solid electrolytes exhibits higher ionic conductivity compared to the amorphous 70Li_2_S–30P_2_S_5_^32,33^. The high ionic conductivity can be explained based on the formation of high-purity crystalline Li_7_P_3_S_11_, which was caused by the high chemical reactivity of the 70Li_2_S–30P_2_S_5_ system in ACN solvent. Commonly, the use of high-DN solvents is an effective strategy for activation of the reaction in a solvent^34–37^. In addition to the dielectric constant, the donor number may also play a role in reactivity.

However, the high reactivity of the Li_2_S–P_2_S_5_ system in low-DN ACN solvent cannot be explained by the DN. In the case of Li_3_PS_4_ synthesized via a solvent, the use of a solvent with low polarity will lead to higher ionic conductivity since solvents with low polarity are more easily removed and yield a lower crystallinity ratio^14,38^. In contrast, the intrinsic high ionic conductivity of Li_7_P_3_S_11_ is caused by a high level of crystallinity^32^, which is explained by the presence of P_2_S_7_ polyhedra in the Li_7_P_3_S_11_ structure^39^. Therefore, a high dielectric constant in the solvent is an essential factor in realizing high conductivity in Li_7_P_3_S_11_. However, the samples using solvents with high dielectric constants other than ACN solvent exhibited ionic insulating properties. The FF, SN, and NM solvents caused a side reaction with the Li_2_S–P_2_S_5_ system, which was responsible for the solvent molecule residue present following heat treatment. Further, in the case of EC, the reactivity of the the Li_2_S–P_2_S_5_ system was low despite it having the highest dielectric constant among the solvents examined. Based on this finding, we believe that differences in the structures of solvent molecules lead to different reactivities with lithium thiophosphates. In addition, high dielectric solvents typically have an extremely high boiling point, which also limits the choice of solvent for the wet chemical synthesis of Li_7_P_3_S_11_ solid electrolytes. The above considerations of the dielectric constant, molecular structure, and boiling point demonstrate that ACN is the most suitable solvent for the liquid-phase synthesis of Li_7_P_3_S_11_ among the investigated solvents.

## Conclusion

In summary, we undertook a systematic study of the effect of solvent on the reactivity of an Li_2_S–P_2_S_5_ system during the liquid-phase synthesis of Li_7_P_3_S_11_ solid electrolytes. The XRD results indicated that Li_7_P_3_S_11_ crystal phase formed in the case of ACN, THP, and Dox solvents. In contrast, a side reaction and insufficient reactivity for the synthesis of Li_7_P_3_S_11_ were confirmed for the CS, NM, FF, SN, and EC solvents. The microstructures of the samples prepared via ACN, THP, and Dox had notable textures, such as a broccoli crown, reflecting the formation process of Li_7_P_3_S_11_ solid electrolytes via a solid-state reaction. TG–DTA indicated that the strength of the chemical interaction between the lithium thiophosphates and solvent molecules within the precursor structure increased in the order of Dox, THP, and then ACN. The ionic conductivities of the samples formed via Dox, THP, and ACN solvent increased in relation to increases in the solvent’s dielectric constant, and the highest ionic conductivity of 0.8 mS cm^–1^ at room temperature was achieved in the sample using ACN solvent. This can be explained based on the formation of high-purity crystalline Li_7_P_3_S_11_, resulting from the high chemical reactivity of the 70Li_2_S–30P_2_S_5_ system in the ACN solvent. These experimental results indicate that the solvent’s polarity, characterized by the dielectric constant, plays an important role in the formation of Li_7_P_3_S_11_ using the liquid-phase method. In addition, a high boiling point and a ring structure that cause steric hindrance were unfavorable solvent attributes for the wet chemical synthesis of Li_7_P_3_S_11_ solid electrolyte. Overall, this study revealed that a solvent’s dielectric constant is a significant factor in obtaining the optimal Li_7_P_3_S_11_ via wet chemical reaction. As it stands, it has been demonstrated that the ACN solvent is most suitable for the liquid-phase synthesis of Li_7_P_3_S_11_ in terms of its dielectric constant, molecular structure, and boiling point.

## Methods

### Synthesis

Lithium sulfide (99.9%, Mitsuwa) and P_2_S_5_ (99%, Merck) were mixed in a mole ratio of 7:3 and then added into Dox (anhydrous, 99.8%, Aldrich), THP (anhydrous, 99%, Aldrich), CS (anhydrous, ≥ 99%, Aldrich), ACN (super dehydrated, 99.8%, Fujifilm), FF (99%, Aldrich), NM (98%, TCI), SN (99.0%, TCI), and EC (anhydrous, 99%, Aldrich) solvents, separately. The mass/volume ratio of the powder/solvent was 1:20 g/ml. The resultant mixtures were stirred at 50 °C for 3 days, and the obtained suspensions were dried under vacuum at 80 °C for 12 h. In the case of FF, SN, and EC, a temperature of 150 °C was required during the drying process. After that, the precursor powders were annealed at 270 °C for 1 h to obtain the Li_7_P_3_S_11_ solid electrolytes. The entire Li_7_P_3_S_11_ synthesis process was handled under an Ar atmosphere.

### Material characterization

Powder XRD measurements were carried out under 2θ = 10° − 50° with a step interval of 0.02° and a scan rate of 1° min^−1^ using a Rigaku Ultima IV diffractometer. The X-ray beam was generated by CuKα radiation (40 kV, 30 mA). We used an XRD holder with a beryllium window (Rigaku). Scanning electron microscopy characterization was carried out using an FE-SEM (S4800, Hitachi), and TG–DTA (EVO II, Rigaku) was performed under Ar flow with a temperature increase of 5 K min^−1^.

### Electrochemical measurements

The total conductivities of the solid electrolytes were measured through samples cold-pressed into pellets with diameters of ~ 10.0 mm under a pressure of 254 MPa. To assemble the cell for electrochemical impedance spectroscopy (EIS) measurements, each sample (~ 80 mg) was filled into a holder made of polyether ether ketone (PEEK) with two stainless steel rods as blocking electrodes. EIS measurements were conducted via alternating-current impedance spectroscopy (SI 1260, Solartron) in a frequency range of 1 MHz to 10 Hz under a dry Ar flow at a temperature of 298 K.

## Supplementary Information

Supplementary Information.
