# Supplementary material for: The effect of solvent on reactivity of the Li2S–P2S5 system in liquid-phase synthesis of Li7P3S11 solid electrolyte
Source: Sci Rep. 2021 Oct 26;11:21097. doi: 10.1038/s41598-021-00662-3 (PMC8548593; doi:10.1038/s41598-021-00662-3)
Supplement: Supplementary file 1 — Supplementary Information. [file 41598_2021_662_MOESM1_ESM.docx]

**SUPPORTING INFORMATION**

**FOR**

**The effect of solvent on reactivity of Li_2_S–P_2_S_5_ system in liquid-phase synthesis of Li_7_P_3_S_11_ solid electrolyte**

Hirotada Gamo, Atsushi Nagai,* Atsunori Matsuda.*

^†^Department of Electrical and Electronic Information Engineering, Toyohashi University of Technology, 1-1 Hibarigaoka, Tempaku-cho, Toyohashi, Aichi 441-8580, Japan

*Corresponding authors: [nagai.atsushi.rn@tut.jp](mailto:nagai.atsushi.rn@tut.jp) (A. Nagai) and matsuda@ee.tut.ac.jp (A. Matsuda)

| 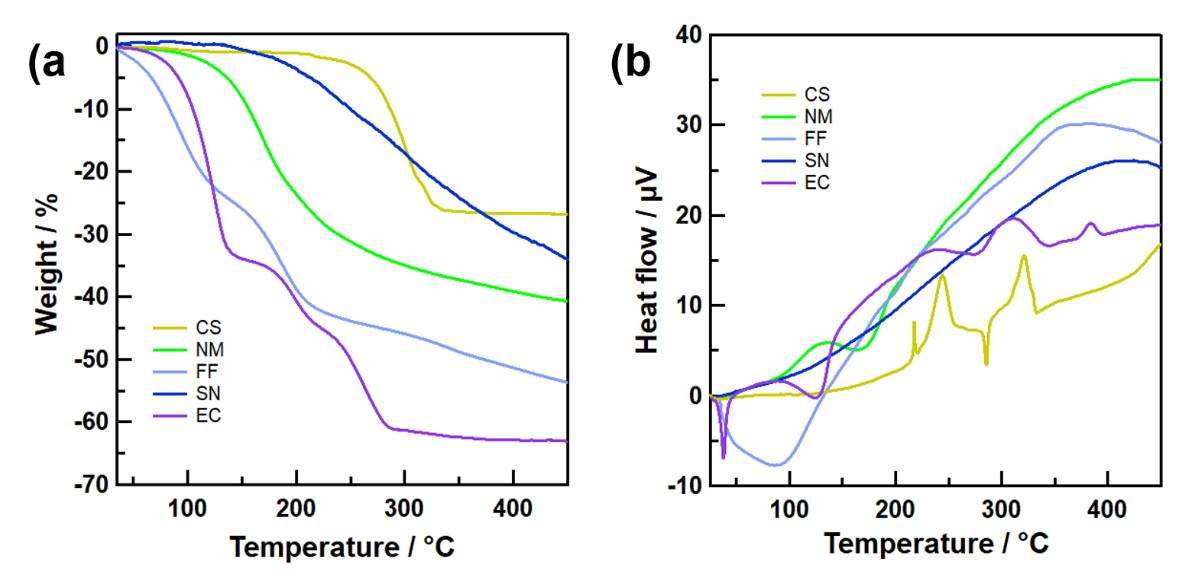 |
| --- |
| Figure S1. (a) TGA curves and (b) DTA curves of 70Li_2_S–30P_2_S_5_ precursors synthesized using CS, FF, NM, SN, and EC solvents. |
